# Homeobox transcription factor muscle segment homeobox 2 (Msx2) correlates with good prognosis in breast cancer patients and induces apoptosis *in vitro*

**DOI:** 10.1186/bcr2621

**Published:** 2010-08-03

**Authors:** Fiona Lanigan, Gabriela Gremel, Rowena Hughes, Donal J Brennan, Finian Martin, Karin Jirström, William M Gallagher

**Affiliations:** 1 University College Dublin School of Biomolecular and Biomedical Science, UCD Conway Institute, University College Dublin, Belfield, Dublin 4, Ireland; 2 Department of Pathology, Mater Misericordiae University Hospital, Eccles Street, Dublin 7, Ireland; 3 Center for Molecular Pathology, Department of Laboratory Medicine, Lund University, Malmö University Hospital, 205 02 Malmö, Sweden

## Abstract

**Introduction:**

The homeobox-containing transcription factor muscle segment homeobox 2 (Msx2) plays an important role in mammary gland development. However, the clinical implications of Msx2 expression in breast cancer are unclear. The aims of this study were to investigate the potential clinical value of Msx2 as a breast cancer biomarker and to clarify its functional role *in vitro*.

**Methods:**

Msx2 gene expression was first examined in a well-validated breast cancer transcriptomic dataset of 295 patients. Msx2 protein expression was then evaluated by immunohistochemistry in a tissue microarray (TMA) containing 281 invasive breast tumours. Finally, to assess the functional role of Msx2 *in vitro*, Msx2 was ectopically expressed in a highly invasive breast tumour cell line (MDA-MB-231) and an immortalised breast cell line (MCF10a), and these cell lines were examined for changes in growth rate, cell death and cell signalling.

**Results:**

Examination of Msx2 mRNA expression in a breast cancer transcriptomic dataset demonstrated that increased levels of Msx2 were associated with good prognosis (*P *= 0.011). Evaluation of Msx2 protein expression on a TMA revealed that Msx2 was detectable in both tumour cell nuclei and cytoplasm. Cytoplasmic Msx2 expression was associated with low grade tumours (*P *= 0.012) and Ki67 negativity (*P *= 0.018). Nuclear Msx2 correlated with low-grade tumours (*P *= 0.015), estrogen receptor positivity (*P *= 0.038), low Ki67 (*P *= 0.005) and high cyclin D1 expression (*P *= 0.037). Increased cytoplasmic Msx2 expression was associated with a prolonged breast cancer-specific survival (*P *= 0.049), recurrence-free survival (*P *= 0.029) and overall survival (*P *= 0.019). Ectopic expression of Msx2 in breast cell lines resulted in radically decreased cell viability mediated by induction of cell death via apoptosis. Further analysis of Msx2-expressing cells revealed increased levels of p21 and phosphorylated extracellular signal-regulated kinase (ERK) and decreased levels of Survivin and the 'split ends' (SPEN) protein family member RBM15.

**Conclusions:**

We conclude that increased Msx2 expression results in improved outcome for breast cancer patients, possibly by increasing the likelihood of tumour cell death by apoptosis.

## Introduction

Homeobox genes are important during embryonic development, where they function to control cell fate and positioning, thereby regulating the morphological development of several organs, including skeletal structures, the heart, teeth, eyes and mammary glands [1-6]. The homeobox family of transcription factors were originally isolated from *Drosophila *and contain a common 61-amino acid domain, known as the homeodomain, which can directly bind DNA and regulate gene transcription [7]. Mutations in the muscle segment homeobox 2 (Msx2) homeodomain which cause loss or gain of Msx2 DNA binding activity can both result in cranial defects [8,9]. Msx2 function can also be affected by subcellular localisation and protein-protein interactions [7].

Within the mammary gland, homeobox genes are thought to be involved in assimilating systemic signals into the precise local interactions required for correct morphogenesis [7,10]. During puberty, the core epithelial structure of the mammary tree is established through the invasion of the terminal end buds from the nipple into the surrounding fat pad [11]. Apoptosis plays an important role in this process, and the level of apoptosis in the mouse pubertal mammary gland is higher than at any other stage of development [12]. In the mouse, Msx2 is expressed during pubertal development and early pregnancy, downregulated during late pregnancy and lactation, and reexpressed during involution [3,13]. Msx2 expression is stimulated by both estradiol and progesterone, and the role of progesterone in promoting branching morphogenesis in the mouse mammary gland is thought to be mediated partially through Msx2 [14]. However, one of the few studies on Msx2 expression in human breast tissues reported a complicated regulation of Msx2 by steroid hormones: Msx2 could be either increased or decreased by steroid hormone treatment, depending on the estrogen/progesterone receptor (ER/PR) status and whether the breast tissue sample was normal or malignant [15].

Given its widespread regulatory role in growth and development, it would not be surprising to find that Msx2 is involved in tumourigenic processes. Indeed, studies in various cell line models have suggested that increased expression of Msx2 can induce neoplastic transformation and epithelial-to-mesenchymal transition (EMT) [16,17]. However, Barnes *et al*. found that Msx2 repressed the activity of the prometastatic bone sialoprotein (BSP) in breast cancer cell lines [18], casting doubt on a proinvasive role for Msx2, as BSP is associated with poor outcome in breast cancer [19]. A role has also been proposed for Msx2 in the regulation of cell death. A recent study of Sonic hedgehog-1 (Shh)-knockout mice [2] found that expression of Msx1 and Msx2 was increased, and this was partially responsible for the massive apoptosis and severe limb defects seen in the Shh-null phenotype. Furthermore, ectopic expression of Msx2 can induce apoptosis in pluripotent murine embryonic carcinoma cells [20], and cranial neural crest-derived cells [5]. This effect on cell death can also be induced by expression of the bone morphogenetic protein (BMP) family: Msx2 and p21 are induced following BMP4 treatment, and both molecules are necessary for BMP4-mediated cell death to occur in several cell types [21,22].

Large-scale clinical studies of Msx2 expression in cancer are few in number. A study of 32 pancreatic adenocarcinomas demonstrated an association between Msx2 expression and high tumour grade and vascular invasion [17]. The only study of Msx2 in breast tumours to date involved four invasive ductal carcinomas and found that Msx2 expression was increased in infiltrating compared to noninfiltrating cells [16]. The prognostic influence of Msx2 expression has not been investigated in a large cohort of breast cancer patients. The aim of this study was to investigate Msx2 expression as a prognostic biomarker in breast cancer. We also sought to clarify the *in vitro *functional role of Msx2. Our findings indicate that increased expression of both Msx2 mRNA and protein are associated with improved outcome in breast cancer. We also found that ectopic expression of Msx2 in breast cell lines leads to induction of apoptosis and radically decreased cell viability. These data suggest that increased Msx2 results in improved outcome for breast cancer patients, possibly by increasing the likelihood of tumour cell death by apoptosis.

## Materials and methods

### Statistical analysis of DNA microarray data

Relevant gene expression and clinical data relating to 295 patients with breast cancer [23] were downloaded from Rosetta Inpharmatics Inc. [24]. The log ratios of gene expression values were used without modification and classified using a previously published method [25]. Tumour samples were classified by first separating into quartiles according to mRNA expression. Adjacent groups with significant overlap of Kaplan-Meier survival curves were combined, and the survival curves of the resulting two groups were compared using the log-rank test. The χ^2 ^test and Fisher's exact test were used for relating mRNA levels to clinicopathological variables.

### Patients

The tissue microarray (TMA) used in this study was derived from a reference cohort of 512 consecutive invasive breast cancer cases diagnosed at Malmö University Hospital, Sweden, between 1988 and 1992, and has been previously described [26,27]. From the original cohort of 512 patients, samples were available from 281 patients for analysis of Msx2 protein expression (this reduced number was primarily due to core loss). Patient and tumour characteristics of the available and missing cohorts are outlined in Additional file 1. The study has been approved by the Ethics Committee at Lund University.

### TMA construction

The TMA was constructed as previously described [25]. Briefly, two 0.6-mm tissue cores were extracted from each donor block using an automated tissue arrayer (MTA-27; Beecher Inc., Sun Prairie, WI, USA) and placed into a recipient block. To confirm Western blot analysis results, a cell pellet array (CPA) was constructed as previously described [27] using formalin-fixed, paraffin-embedded (FFPE) breast cell lines.

### Cell culture

All cell lines (MCF-7, T47 D, BT474, ZR75-1, MDA-MB-231, and SKBR3) were purchased from the European Collection of Cell Cultures (Wiltshire, UK), except for the MCF10a cell line, which was a gift from Dr. Geert Berx, University of Ghent, Belgium, and the Hs578t isogenic cell line series (Hs578t P and i8) [28], which was a gift from Dr. Susan McDonnell, University College Dublin. All cell lines were maintained as previously described [27].

### Western blot analysis

For analysis of cell lines, protein was extracted as previously described [27]. Lysates were separated by reducing sodium dodecyl sulphate-polymerase gel electrophoresis (SDS-PAGE), transferred to polyvinylidene fluoride (PVDF), and immunoblotted using antibodies against Msx2 (clone 2E12, 1:1,000; Abcam, Cambridge, UK), extracellular signal-regulated kinase (ERK) (1:1,000; Santa Cruz Biotechnology, Santa Cruz, CA, USA), p-ERK (1:1,000; Santa Cruz Biotechnology), p21 (1:1,000; BD Biosciences, Franklin Lakes, NJ, USA), Survivin (1:100; Santa Cruz Biotechnology), cyclin D1 (1:1,000; Santa Cruz Biotechnology), DLX5 (clone 3B11; 1:1,000; Abnova, Taipei, Taiwan), Twist (1:1,000; Santa Cruz Biotechnology), and Smad4 (1:1,000; Cell Signaling, Danvers, MA, USA). Membranes were stripped and reprobed with anti-β-actin (1:5,000 dilution; Abcam) as a loading control.

### Immunohistochemistry

TMA and CPA sections (4 μm) were rehydrated in descending gradient alcohols. Heat-mediated antigen retrieval was performed using 10 mM sodium citrate buffer (pH 6) in a PT module (LabVision; Thermo-Fisher Scientific, Fremont, CA, USA) for 15 min at 95 °C, followed by immunohistochemistry (IHC) in a Lab Vision Autostainer 360 (LabVision, Fremont, CA, USA) for Msx2 (clone 2E12; 1:25; Abcam) or in the Ventana Benchmark system (Ventana Medical Systems Inc., Oro Valley, AZ) using prediluted antibodies to estrogen receptor (clone 6F11; Ventana Medical Systems Inc.), progesterone receptor (clone 16; Ventana Medical Systems Inc.) and Her2 (Pathway, Clone CB-11, Ventana Medical Systems) or in the Dako Techmate 500 system (Dako, Glostrup, Denmark) for Ki-67 (M7240, 1:200; Dako) and cyclin D1 (clone DSC-6, 1:100; Dako). A mouse IgG2a isotype control (Abcam) was used to evaluate Msx2 antibody specificity.

Slides were scanned at ×20 magnification using a ScanScopeXT slide scanner (Aperio Technologies, Vista, CA, USA). Tumour samples were evaluated by two independent observers (RH and FL) and scored for Msx2 staining intensity in both the cytoplasmic and nuclear compartments on a scale of 0 to 3, where 0 is negative, 1 is weakly positive, 2 is medium positive and 3 is strongly positive. Nuclear staining for Msx2 was found to be relatively homogeneous within tumour samples, and thus the percentage of nuclear staining was not included in the scoring system. The mean value of both scores for each patient was used for statistical analysis. ER, PR, HER2 and Ki-67 were assessed as previously described [29].

To control for the subjectivity inherent in the manual scoring process, we utilised a co-localisation image analysis algorithm (Aperio Technologies) to further examine Msx2 localisation and expression. This algorithm classifies each pixel as either blue (negative nuclear), blue and brown (positive nuclear), or brown (positive cytoplasmic). The average positive pixel intensities of 3,3'-diaminobenzidine (DAB) staining in the cytoplasm and nuclei were used for statistical analysis, divided at the 50th percentile.

### Statistical analysis of TMA data

The χ^2 ^test and Fisher's exact test were used to evaluate associations between Msx2 expression and clinicopathological characteristics. Kaplan-Meier plots were used for survival analysis, and the log-rank test was used to compare curves separated according to Msx2 expression. Cox proportional hazards regression was used to estimate hazard ratios (HR). All calculations were carried out using SPSS version 15 (SPSS Inc., Chicago, IL, USA).

### Lentiviral overexpression of Msx2

Human embryonic kidney (HEK)-293t cells, at ~60-70% confluence, were transfected using a calcium phosphate transfection method with an LLCIEP vector (Trono Laboratory, Lausanne, Switzerland) with no insert (EV) or containing full-length Msx2 with a V5 tag (MSX2), together with packaging and envelope plasmids (PSPAX2 and PMD2G; Trono Laboratory). The media were refreshed after 6-8 hr and, after a further 48 hr, the viral supernatant was removed and filtered through a 0.45-μm low-protein binding filter (Millipore, Billerica, MA, USA). This was added to ~50% confluent MDA-MB-231 or MCF10a cells at a 1:3 dilution with fresh media, along with 8 μg/ml polybrene. MCF10a cells were centrifuged at 300 × *g *for 1 hr at 30°C following addition of the viral supernatant. Media were refreshed after 24 hr. The transduction efficiency was estimated at >95% using immunofluorescence microscopy for Msx2 expression.

### Immunofluorescence studies

Cells growing on chamber slides (Nunc, Roskilde, Denmark) were fixed with 3.7% paraformaldehyde for 20 min, permeabilised with 0.5% Triton-X 100 for 10 min, and labelled immunofluorescently using an anti-Msx2 mouse monoclonal antibody (Abcam), followed by incubation with a rhodamine-coupled secondary antibody (Abcam) and 4',6-diamidino-2-phenylindole (DAPI) (Sigma-Aldrich, Dorset, UK). Images were obtained using a Zeiss LSM-510-Meta confocal microscope.

### MTT proliferation assay

Cellular proliferation was measured using an MTT (3-(4,5-dimethylthiazol-2-yl)-2,5-diphenyltetrazolium bromide) colorimetric assay over a period of 5 days. Cells were seeded at a density of 2,000 cells/well in 96-well plates, with one plate measured every day for 5 days. Cells were incubated with MTT reagent (5 mg/ml) for 4 hr, solubilised in dimethyl sulfoxide, and the absorbance at 570 nm was measured.

### Colony formation assay

Cells were seeded at a density of 500 cells/well in a six-well plate. Plates were incubated for 3 weeks with media changed weekly. Cells were fixed for 15 min in 10% neutral-buffered formalin and stained with 0.25% crystal violet solution. Colonies were counted by eye, with three wells counted per cell line in each replicate experiment.

### Apoptosis assay

The Caspase-Glo 3/7 kit (Promega, Madison, WI, USA) was used to determine levels of apoptosis in cell lines. Cells were seeded in two 96-well plates at 5,000 cells/well and allowed to grow for 2 days. Media were removed from one plate, replaced with Caspase-Glo working solution in a 1:1 dilution to normal media, left at room temperature for 1 hr, and read using a luminescent plate reader (GloMax Multi Detection System, Promega). The cells on the second plate were trypsinized and counted to normalize the luminescent signal to cell number.

### Cell cycle analysis

Cells at ~60-70% confluence were harvested and washed in ice-cold phosphate-buffered saline (PBS) before fixation for 1 hr in ice-cold 70% ethanol in PBS. Cells were collected by centrifugation, washed in PBS and recentrifuged before resuspension in 1 ml of ice-cold PBS containing 0.4 mg/ml propidium iodide (PI). Cells were left for 30 min on ice prior to flow cytometry analysis on a Coulter Epic XL flow cytometer (Beckman Coulter Inc., Brea, CA, USA), to measure the PI uptake and thus the DNA content of the cells.

## Results

### Msx2 mRNA expression is associated with good prognosis in a DNA microarray dataset

**Figure 1:**
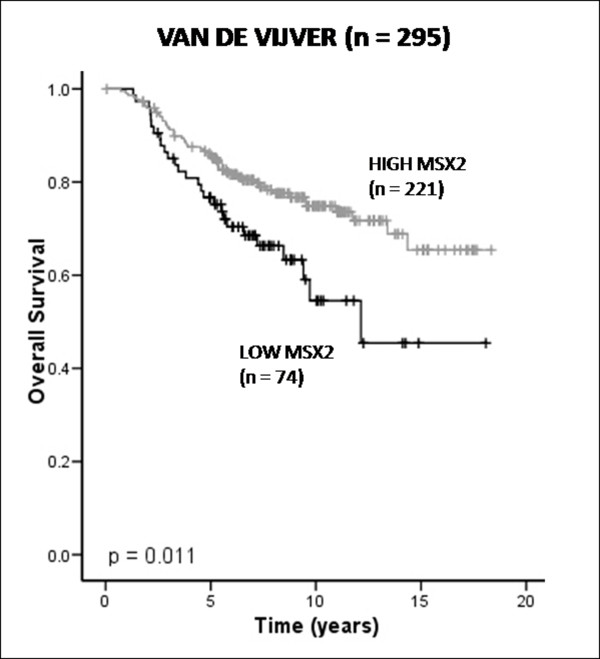
**Analysis of muscle segment homeobox 2 (Msx2) expression at the mRNA level**. Kaplan-Meier estimates of overall survival (OS) stratified by Msx2 expression within the Van de Vijver dataset of 295 breast tumours.

Expression levels of Msx2 mRNA were analysed in a publicly available DNA microarray dataset derived from 295 primary invasive breast tumours [23]. Using a previously described method [25], 74 tumours were classified as expressing low levels of Msx2 mRNA, and 221 tumours were classified as expressing high levels of Msx2 mRNA, based on the log ratios of gene expression values. Survival analysis of these tumours revealed that increased expression of Msx2 mRNA was associated with good prognosis (*P *= 0.011) (Figure 1). Univariate Cox regression analysis of Msx2 mRNA expression as a continuous variable supported this association with favourable outcome (HR 0.47; 95% CI 0.24-0.95; *P *= 0.035). Associations between Msx2 mRNA expression and commonly used clinical biomarkers were also examined (Table 1). Increased Msx2 mRNA expression correlated with ER-positive (*P *< 0.001) and low-grade (*P *= 0.003) tumours; moreover, it was associated with good prognosis according to the 70-gene signature defined by van't Veer *et al*. [30] (*P *< 0.001). In relation to breast cancer molecular subtypes described by Sorlie *et al*. [31], Msx2 mRNA expression was particularly low in the basal subtype and high in the luminal B and Her2 subtypes of breast cancer (*P *= 0.001).

**Table 1:** Analysis of Msx2 expression at the mRNA level within the van de Vijver dataset^a^

Variable	Van de Vijver data set (*n *= 295)		
	Low Msx2 (*n *= 74)	High Msx2 (*n *= 221)	*P *value
Age			0.592
≤50	65 (87.8)	199 (90)	
> 50	9 (12.2)	22 (10)	
Tumour size			0.297
≤2 cm	35 (47.3)	120 (54.3)	
> 2 cm	39 (52.7)	101 (45.7)	
Nodal status			0.056
Negative	45 (60.8)	106 (48)	
Positive	29 (39.2)	115 (52)	
Tumour grade			0.003
Low	12 (16.2)	63 (28.5)	
Intermediate	20 (27)	81 (36.7)	
High	42 (56.8)	77 (34.8)	
ER status			<0.001
Negative	39 (52.7)	30 (13.6)	
Positive	35 (47.3)	191 (86.4)	
Tumour subtype			<0.001*
Normal	7 (9.5)	24 (10.9)	
Luminal A	20 (27)	68 (30.8)	
Luminal B	11 (14.9)	70 (31.7)	
Basal	30 (40.5)	16 (7.2)	
Her2	6 (8.1)	43 (19.5)	
70-gene signature			0.003
Poor	56 (75.7)	124 (56.1)	
Good	18 (24.3)	97 (43.9)	

^a^ER, estrogen receptor; *Fisher's exact test;, otherwise, χ2 test.

### Msx2 protein expression in primary breast tumours

**Figure 2:**
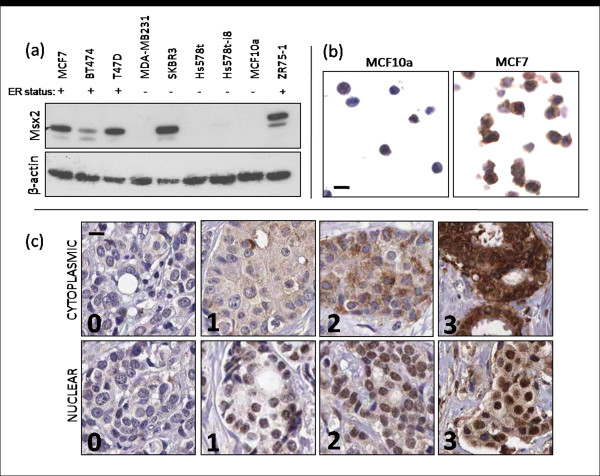
**Validation of Msx2 antibody specificity**. **(a)** Western blot analysis of breast cell lines for Msx2 expression, showing Msx2 migrating at approximately 37 kDa. β-actin levels were used to evaluate protein loading. **(b)** Immunohistochemical staining for Msx2 on formalin-fixed, paraffin-embedded (FFPE) breast cell lines (×20 magnification). **(c)** Immunohistochemical staining for Msx2 on breast tumour tissue microarray (TMA) cores, showing examples of tumours with scores from 0 to 3 for nuclear and cytoplasmic staining (×20 magnification; scale bar represents 10 μm).

The specificity of the Msx2 antibody was validated first by Western blot analysis on a panel of breast cancer cell lines (Figure 2a) and second by IHC in FFPE breast cancer cell lines (Figure 2b). High Msx2 expression was detected by Western blot analysis in MCF7, T47 D, SKBR3 and ZR75-1 cell lines, with low expression seen in BT474 cells and no detectable expression in MDA-MB231, Hs578t, Hs578t-i8 and MCF10a cell lines. This expression pattern appears for the most part to correlate with ER status, apart from the high expression of Msx2 seen in the ER-negative cell line SK-BR3. MCF7 and MCF10a cell lines were used as positive and negative controls, respectively, for all further IHC procedures. Msx2 protein expression was assessed using IHC in a breast cancer TMA constructed from a cohort of 512 patients. Owing to core loss, it was possible to evaluate Msx2 protein expression in 281 tumours (55%) of the 512 tumours represented on the TMA. To evaluate our study for any potential selection bias, baseline clinicopathological characteristics from both the evaluated or 'Msx2 known' cohort (*n *= 281) and the unevaluated or 'Msx2 unknown' cohort (*n *= 231) are presented in Additional file 1. No difference was seen in the expression of any variable between either cohort.

Tumour-specific nuclear and cytoplasmic expression of Msx2 protein was evident, with levels of Msx2 in these subcellular compartments being scored separately (Figure 2c). Staining in each compartment was scored on a scale from 0 to 3 on the basis of staining intensity. To divide the tumours into two relatively equal-sized groups, high cytoplasmic Msx2 was defined as a staining intensity ≥2, and high nuclear Msx2 was defined as a staining intensity ≥1. On the basis of this analysis, 168 tumours (59.8%) were classified as expressing low levels of cytoplasmic Msx2, and 113 tumours (40.2%) were classified as expressing high cytoplasmic Msx2. Similarly, 122 tumours (43.4%) were classified as expressing low nuclear Msx2, and 159 tumours (56.6%) were classified as expressing high nuclear Msx2.

Associations between nuclear and cytoplasmic Msx2 protein expression and a number of well-established clinicopathological variables were then investigated (Table 2). Cytoplasmic Msx2 expression was associated with low-grade tumours (*P *= 0.012) and low expression of Ki67 (proliferation-related Ki-67 antigen) (*P *= 0.018). Nuclear Msx2 expression was associated with low-grade (*P *= 0.015), ER-positive (*P *= 0.038) tumours, low Ki67 expression (*P *= 0.005), and increased nuclear cyclin D1 expression (*P *= 0.037).

**Table 2:** Association of cytoplasmic and nuclear Msx2 with clinicopathological parameters in 281 tumours on a TMA^a^

Variable	Cytoplasmic Msx2			Nuclear Msx2		
	Low Msx2 (*n *= 168)	High Msx2 (*n *= 113)	*P *value	Low Msx2 (*n *= 122)	High Msx2 (*n *= 159)	*P *value
Age						
Median (range)	66 (35-96)	64 (36-89)		65 (35-96)	65.5 (35-91)	
≤50	23 (13.7)	14 (12.4)	0.752	19 (15.6)	18 (11.3)	0.296
>50	145 (86.3)	99 (87.6)		103 (84.4)	141 (88.7)	
Tumour size						
Median (range)	18 (0-100)	16 (1-100)		19 (0-100)	15.5 (1-100)	
≤2 cm	105 (62.5)	75 (66.4)	0.507	74 (60.7)	106 (66.7)	0.298
>2 cm	63 (37.5)	38 (33.6)		48 (39.3)	53 (33.3)	
Histological type						
Ductal	115 (75.7)	75 (69.4)	0.157*	80 (72.7)	110 (73.3)	0.715*
Lobular	19 (12.5)	22 (20.4)		19 (17.3)	22 (14.7)	
Tubular	9 (5.9)	8 (7.4)		5 (4.5)	12 (8)	
Medullary	5 3.3)	0		3 (2.7)	2 (1.3)	
Mucinous	4 (2.6)	3 (2.8)		3 (2.7)	4 (2.7)	
Unknown	16	5		12	9	
Nodal status						
Negative	89 (59.3)	69 (69)	0.121	66 (61.1)	92 (64.8)	0.550
Positive	61 (40.7)	31 (31)		42 (38.9)	50 (35.2)	
Unknown	18	13		14	17	
Tumour grade						
I	34 (20.4)	35 (31)	0.012	20 (16.4)	49 (31)	0.015
II	66 (39.5)	51 (45.1)		54 (44.3)	63 (39.9)	
III	67 (40.1)	27 (23.9)		48 (39.3)	46 (29.1)	
Unknown	1	0		0	1	
ER status						
Negative	23 (14.1)	16 (14.7)	0.896	23 (19.3)	16 (10.5)	0.038
Positive	140 (85.9)	93 (85.3)		96 (80.7)	137 (89.5)	
Unknown	5	4		3	6	
PR status						
Negative	47 (36.4)	38 (40.9)	0.503	41 (40.6)	44 (36.4)	0.518
Positive	82 (63.6)	55 (59.1)		60 (59.4)	77 (63.6)	
Unknown	39	20		21	38	
Ki 67 (%)						
<10%	56 (34.1)	52 (48.6)	0.018	35 (30.2)	73 (47.1)	0.005
>10%	108 (65.9)	55 (51.4)		81 (69.8)	82 (52.9)	
Unknown	4	6		6	4	
VEGF (%)						
Low (0-2+)	92 (78)	77 (86.5)	0.116	76 (83.5)	93 (80.2)	0.537
High (3)	26 (22)	12 (13.5)		15 (16.5)	23 (19.8)	
Unknown	50	24		31	43	
Her2 (%)						
Low (0-2)	141 (89.2)	95 (92.2)	0.422	100 (89.3)	136 (91.3)	0.589
High (3)	17 (10.8)	8 (7.8)		12 (10.7)	13 (8.7)	
Unknown	10	10		10	10	
Cyclin D1 (%)						
Low (0-1%)	20 (12.5)	17 (15.7)	0.706	21 (17.9)	16 (10.6)	0.037
Med (2-25%)	109 (68.1)	69 (63.9)		80 (68.4)	98 (64.9)	
High (>25%)	31 (19.4)	22 (20.4)		16 (13.7)	37 (24.5)	
Unknown	8	5		5	8	

^a^Msx2, muscle segment homeobox 2; ER, estrogen receptor; PR, progesterone receptor; Ki67, proliferation-related Ki-67 antigen; VEGF, vascular endothelial growth factor; Her2, human epidermal growth factor 2; *Fisher's exact test;, otherwise, χ2 test.

### Increased Msx2 protein expression is associated with prolonged patient survival

**Figure 3:**
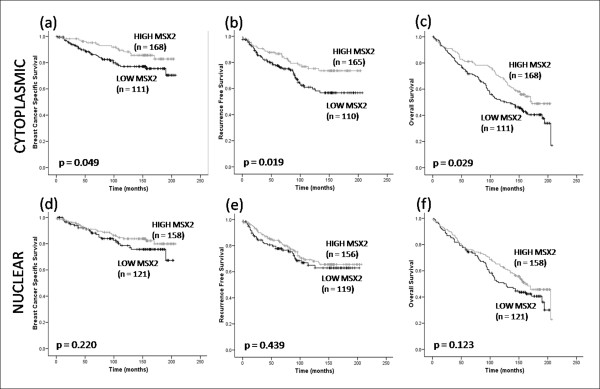
**Kaplan-Meier estimates of survival in 281 breast tumours on a TMA**. **(a)** Breast cancer-specific survival (BCSS), **(b)** recurrence-free survival (RFS), **(c)** OS stratified according to cytoplasmic Msx2 expression. **(d)** BCSS, **(e)** RFS, and **(f)** OS stratified according to nuclear Msx2 expression.

The relationship between Msx2 protein expression and survival was then examined. In agreement with our previous findings, cytoplasmic Msx2 was associated with longer breast cancer-specific survival (BCSS) (*P *= 0.049), recurrence-free survival (RFS) (*P *= 0.029), and overall survival (OS) (*P *= 0.019) (Figure 3). In contrast, nuclear Msx2 expression was not associated with outcome at any endpoint: BCSS (*P *= 0.220), RFS (*P *= 0.439) or OS (*P *= 0.123). Univariate Cox regression analysis confirmed that increased cytoplasmic Msx2 expression was associated with an extended BCSS (HR 0.54; 95% CI 0.29-1.01; *P *= 0.053), RFS (HR 0.57; 95% CI 0.35-0.92; *P *= 0.021) and OS (HR 0.69; 95% CI 0.49-0.97; *P *= 0.031). To compare the prognostic impact of Msx2 with well-established clinical variables, a multivariate Cox regression analysis was carried out using OS as an endpoint (Table 3). Cytoplasmic Msx2 expression was an independent predictor of prolonged OS (HR 0.58; 95% CI 0.36-0.93; *P *= 0.023), along with tumour grade (HR 1.78; 95% CI 1.06-2.99; *P *= 0.028) and nodal status (HR 3.06; 95% CI 1.95-4.80; *P *< 0.001). Univariate Cox regression analysis of nuclear Msx2 showed no association with outcome (HR 0.78; 95% CI 0.56-1.07; *P *= 0.124).

**Table 3:** Cox regression analysis of overall survival in the entire patient cohort^a^

	Entire cohort (*n *= 281)					
	Univariate			Multivariate*		
Prognostic factor	HR	(95% CI)	*P *value	HR	(95% CI)	*P *value
Cytoplasmic Msx2 (high vs. low, ref)	0.69	0.49-0.97	0.031	0.58	0.36-0.93	0.023
Age (continuous)	1.06	1.05-1.07	<0.001	1.05	1.03-1.07	<0.001
Tumour size (continuous)	1.01	1.00-1.01	<0.001	1.01	1.00-1.02	0.006
Tumour grade (3 vs. 0-2, ref)	2.09	1.63-2.67	<0.001	1.78	1.06-2.99	0.028
Nodal status (pos vs. neg, ref)	3.18	2.43-4.16	<0.001	3.06	1.95-4.80	<0.001
ER (pos vs. neg, ref)	0.73	0.52-1.02	0.062	0.92	0.38-2.21	0.855
PR (pos vs. neg, ref)	0.61	0.46-0.81	0.001	0.68	0.41-1.13	0.140
Her2 (3 vs. 0-2, ref)	0.77	0.49-1.22	0.270	0.63	0.23-1.69	0.355
Ki-67 (>10% vs. <10%, ref)	1.31	1.01-1.71	0.044	1.15	0.67-1.97	0.610

^a^Msx2, muscle segment homeobox 2; ER, estrogen receptor; PR, progesterone receptor; Her2, human epidermal growth factor 2; Ki67, proliferation-related Ki-67 antigen. *Adjusted for all other variables in the table; ref, referent group.

To control for subjectivity in the manual scoring process, we used a co-localisation algorithm (Aperio) to impartially examine Msx2 expression and localisation. We found a strong correlation between automated and manual scores (Spearman's ρ = 0.745; *P *< 0.001). Using the automated data, cytoplasmic Msx2 expression was associated with overall survival (*P *= 0.008), whereas nuclear Msx2 expression was not (*P *= 0.097), in agreement with manual scoring data (Additional file 2).

### Msx2 induces cell death by apoptosis in both transformed and immortalized breast cell lines

**Figure 4:**
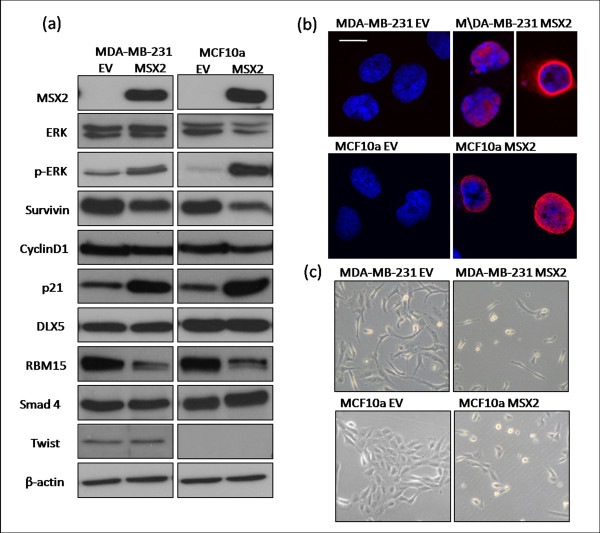
**Characterisation of Msx2-overexpressing breast cell lines**. **(a)** Light microscopic images of MDA-MB-231 and MCF10a cell lines, transduced with EV or Msx2 expression vector, showing increased cell death in Msx2-overexpressing cells. **(b)** Immunofluorescence microscopy detecting Msx2 localisation in MDA-MB-231 and MCF10a cell lines with ectopic Msx2 expression. Scale bar represents 10 μm. Images are representative of three experiments. **(c)** Examination of a number of cell cycle and Msx2-interacting proteins by Western blot analysis in empty vector and Msx2-overexpressing MDA-MB-231 and MCF10a cell lines. Images are representative of three replicates.

Having shown that Msx2 expression correlates with good prognosis in breast tumours, we proceeded to examine the role of Msx2 *in vitro*. Cells suitable for Msx2 overexpression were determined by Western blot analysis (Figure 2a). MDA-MB-231, Hs578t, and MCF10a cells all had undetectable levels of Msx2 protein. We chose a highly invasive breast cancer cell line (MDA-MB-231) and an immortalized mammary epithelial cell line (MCF10a) for our study. Ectopic Msx2 was overexpressed in these cell lines, and expression was verified by Western blot analysis, which showed that both the MDA-MB-231-Msx2 (designated MDA-Msx2) and MCF10a-Msx2 cell lines displayed high levels of Msx2 protein compared to the corresponding empty vector (EV) controls (Figure 4a). We also examined these cell lines by immunofluorescence microscopy using a fluorescently tagged secondary antibody (Figure 4b). This revealed high levels of Msx2 in both MDA-Msx2 and MCF10a-Msx2 cell lines, the expression of which was predominantly nuclear and perinuclear.

**Figure 5:**
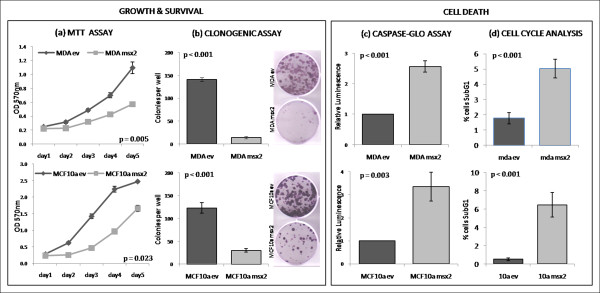
**Functional analysis of Msx2-overexpressing breast cell lines**. **(a)** Measurement of cell proliferation rates by MTT assay. Significance was determined by comparing the slopes of the lines of best fit. **(b)** Measurement of colony forming ability of cellsby clonogenic assays. **(c)** Measurement of caspase-3 and -7 activation as an indicator of apoptosis. Values are relative to control levels. **(d)** Cell cycle analysis by propidium iodide staining. Values represent the percentage of cells within sub-G1 gate. For all assays, values denote the mean readings of three independent replicates (*n *= 3 within each experiment), and error bars represent SD within replicate experiments. Unpaired, two-tailed Student's *t*-tests were used to determine the significance of difference between samples.

We also observed that Msx2-expressing cell lines appeared to be undergoing cell death, whereas the EV-transduced cells grew unperturbed (Figure 4c); thus, the effect of Msx2 overexpression on cell growth, anchorage-independent growth, and cell death was examined (Figure 5). A significant reduction in growth rates, as measured by MTT assay over a period of 5 days, was observed between EV-transduced cells and their Msx2-overexpressing counterparts, namely, MDA-Msx2 (*P *= 0.005) and MCF10a-Msx2 (*P *= 0.023) (Figure 5a). Clonogenic assays also revealed a dramatic decrease in cell viability in both MSX2-expressing cell lines (*P *< 0.001 for both) (Figure 5b). The mechanism of cellular death was then investigated using a luminescence-based assay (Caspase-Glo 3/7), which detects apoptosis by measuring levels of caspase-3 and -7 within cells. This revealed a significant increase in caspase activity in both MDA-Msx2 (*P *< 0.001) and MCF10a-Msx2 (*P *= 0.003) cell lines relative to controls, indicating that the mode of cell death was apoptosis (Figure 5c). The proportion of cells in sub-G1, as measured by flow cytometric analysis, was also significantly higher in MDA-Msx2 (*P *< 0.001) and MCF10a-Msx2 (*P *< 0.001) cell lines compared to controls (Figure 5d). Taken together, these data indicate that increased levels of Msx2 in breast cells can lead to induction of cell death by apoptosis.

### Msx2 overexpression in cell line models leads to an alteration in a number of signalling pathways

To elucidate the mechanism by which Msx2 might induce apoptosis, the expression of several proteins involved in cell signalling was examined in the Msx2-overexpressing and control cells (Figure 4a). We first examined ERK activation and found that ectopic Msx2 expression induced activation of ERK by phosphorylation at Tyr204 in both the MDA-Msx2 and MCF10a-Msx2 cell lines compared to EV controls. We proceeded to look at a number of proteins which regulate cell cycle and apoptosis and found that the Msx2-overexpressing cell lines had notably increased levels of the cyclin-dependent kinase inhibitor p21 (Cip1) and decreased levels of the apoptotic inhibitor Survivin (BIRC5). Levels of cyclin D1 protein were unaffected by ectopic Msx2 expression in these cell lines.

We next focused on proteins involved in transcriptional regulation which may interact with Msx2 and affect its activity as a transcription factor. Levels of the homeobox transcription factor, DLX5, were unaffected by Msx2 overexpression, as was the transcriptional regulator Smad4. Msx2 is known to interact with the SPEN (a derivation of 'split ends' in *Drosophila*) family of transcriptional regulators [7,32], and we found that levels of RBM15 (RNA-binding motif protein 15), a member of the SPEN family, were decreased in Msx2-overexpressing cells. Finally, we looked at expression of Twist, a transcription factor known to promote EMT, which has previously been shown to be induced by Msx2 [17]. Twist was expressed in the transformed MDA-MB-231 cell line, but not in the immortalized MCF10a cell line. We saw no change in Twist expression between control and Msx2-overexpressing cells.

### Msx2 mRNA expression correlates with expression of cell cycle proteins in tumour samples

To correlate our *in vitro *cell line data with the clinical situation, we examined the expression of a number of cell survival, cell cycle and Msx2-interacting genes within the previously described Van de Vijver dataset [23] (Table 4). In relation to cell survival, high levels of Msx2 mRNA were associated with low mRNA levels of the apoptotic inhibitor Survivin (*P *< 0.001). With regard to cell cycle, increased Msx2 mRNA expression was associated with high cyclin D1 (*P *< 0.001) and low levels of the cyclins A1 (*P *= 0.037), A2 (*P *= 0.007), B2 (*P *= 0.014), and E1 (*P *= 0.003). We also examined the Msx2-interacting protein, Dlx5, which was associated with poor prognosis (*P *= 0.032) but did not significantly correlate with Msx2 expression (*P *= 0.077). In addition, we found that high Msx2 expression was associated with increased levels of BMP4 (*P *= 0.017), which has previously been shown to induce Msx2 expression [21]. BMP4 alone was associated with good prognosis (*P *< 0.001). Expression data on p21 or RBM15 was not available within this dataset.

**Table 4:** Association of Msx2 with cell cycle-associated and Msx2-interacting genes at the mRNA level within the Van de Vijver dataset^a^

Variable	Van de Vivjer data set (*n *= 295)			
	Low Msx2 (*n *= 74)	High Msx2 (*n *= 221)	*P *value	Association with Prognosis?
Survivin			<0.001	Poor**
Low	24 (32.4)	125 (56.6)		
High	50 (67.6)	96 (43.4)		
Cyclin D1			<0.001	NS
Low High	32 (43.2) 42 (56.8)	42 (19) 179 (81)		
Cyclin A1			0.037	Poor**
Low	49 (66.2)	173 (78.3)		
High	25 (33.8)	48 (21.7)		
Cyclin A2			0.007	Poor**
Low	27 (36.5)	121 (54.8)		
High	47 (63.5)	100 (45.2)		
Cyclin B2			0.014	Poor**
Low	28 (37.8)	120 (54.3)		
High	46 (62.2)	101 (45.7)		
Cyclin E1			0.003	Poor**
Low	46 (62.2)	175 (79.5)		
High	28 (37.8)	45 (20.5)		
DLX5			0.077	Poor*
Low	50 (67.6)	172 (77.8)		
High	24 (32.4)	49 (22.2)		
BMP4			0.017	Good**
Low	46 (62.2)	102 (46.2)		
High	28 (37.8)	119 (53.8)		

^a^BMP, bone morphogenetic protein; DLX5, distal-less homeobox 5; *P < 0.05 **P < 0.01 (log rank test); otherwise χ2 test.

## Discussion

The objective of this study was to clarify the role of Msx2 in breast cancer. Previous studies have postulated a role for Msx2 in the advancement of the invasive phenotype in breast cancer [16]. However, it has become clear from our analysis of two independent patient cohorts that increased Msx2 mRNA and protein expression is associated with improved outcome in breast cancer. Interestingly, in the TMA-based study, cytoplasmic as opposed to nuclear MSX2 expression was more significant in relation to improved survival, suggesting that the DNA-binding activity of Msx2 may not be the main reason for the beneficial effects of Msx2 overexpression. However, some factors which were not associated with cytoplasmic Msx2, such as ER status and cyclin D1, were associated with nuclear Msx2, indicating that Msx2 may have some additional activity upon translocation to the nucleus.

Our hypothesis that Msx2 is a good prognostic marker in breast cancer was further supported by the subsequent *in vitro *investigations, when we overexpressed Msx2 in both an immortalized mammary epithelial cell line (MCF10a) and an invasive breast cancer cell line (MDA-MB-231). In both cell lines, a drastic reduction in cell viability, apparently due to induction of apoptosis, was observed. Investigation of how this apoptosis might occur through dissection of signalling pathways showed that ERK activation was increased in Msx2-overexpressing cells. Although ERK activation is known to stimulate cell growth in many situations, it can also induce apoptosis in certain cases, depending on the downstream signals activated [33]. We also found an increase in p21 expression concurrent with Msx2 in these cell lines. Previous studies of BMP4-induced apoptosis in various cell types [21,22] have shown that both Msx2 and p21 are induced following BMP4 treatment. Inhibition of either molecule alone was sufficient to block cell death, suggesting that both are needed for apoptosis to occur and that they may be part of the same apoptotic cascade. Indeed, the BMP pathway may be involved in modulating the effect of Msx2 expression in breast cancer: analysis of BMP4 expression in 295 tumours from a transcriptomic dataset revealed that Msx2 is associated with high levels of BMP4 in breast tumours, and BMP4 itself was associated with improved patient outcome within this dataset.

In-depth analysis of cell cycle machinery within Msx2-overexpressing cells showed that levels of the apoptosis inhibitor Survivin were decreased, possibly leaving these cells susceptible to apoptosis. Survivin is a significant predictor of poor prognosis in breast cancer [34]. Although we saw no change in levels of cyclin D1 following Msx2 overexpression *in vitro*, when we examined the van de Vijver dataset, we found that Msx2 was associated with high cyclin D1 and low cyclin A1, A2, B2 and E1. High cyclin D1 is needed for cells to pass the G1 checkpoint, and it was previously thought to be associated with increased proliferation and poor clinical outcome. However, further studies have revealed that cyclin D1 can be associated with improved outcome in breast cancer, possibly due to the blocking effects of p27, which is often increased in parallel with cyclin D1 [35,36]. In this transcriptomic dataset, cyclin D1 was associated with good prognosis and also with p27 expression (*P *< 0.001), indicating a possible mechanism whereby high expression of Msx2 can be linked to cell cycle arrest. The fact that Msx2 mRNA expression in these tumours correlates with markers of cell cycle arrest and apoptosis suggests that the apoptosis which we observed *in vitro *may represent a real phenomenon in breast tumours *in vivo*. However, further mechanistic analyses are needed to confirm this hypothesis.

Further analysis of the Msx2-overexpressing cell lines revealed that levels of the SPEN family member RBM15 were downregulated concurrently with Msx2 overexpression. Although very little is known about the physiological role of RBM15, it is closely related to SHARP, another member of the SPEN family which is known to interact with Msx2 [32]. This family of proteins are characterized by N-terminal RNA recognition motifs and a conserved SPOC (SPEN paralog and ortholog C-terminal) domain [37]. The conserved SPOC domain in both SHARP and RBM15 is known to interact with SMRT (silencing mediator of retinoid and thyroid hormone receptors) and NCoR (nuclear receptor corepressor) corepressors to mediate transcriptional repression [38]. RBM15 is also involved in regulating activation of Notch signalling [39], nuclear export of mRNA [40], and hematopoietic development and cell fate [41]. Deletion of RBM15 in mice is embryonic lethal, and conditional deletion of RBM15 in adult mice results in a reduction in levels of white blood cells [41]. Although the mechanism of action of RBM15 is unclear, the downregulation of this protein in Msx2-overexpressing cell lines indicates that RBM15 may interact directly or indirectly with Msx2 and could mediate, in part, the effects of Msx2 overexpression.

Perhaps it is the background level of Msx2 interacting proteins and cofactors that determines the effect of increased levels of Msx2 in breast tumours. A link with the ER signalling pathway is also likely, as previous studies have shown that Msx2 expression can be induced in breast cancer cell lines and breast explants following estradiol or progesterone treatment [14,15]. We observed a correlation between Msx2 expression and ER status in the majority of the cell lines that were examined in this study (Figure 2a). The only previous study of Msx2 overexpression in the breast found an association between Msx2 overexpression and induction of EMT and cellular invasion [16], with no evidence of a reduction in cell viability or induction of apoptosis. However, the earlier study used mouse mammary epithelial cells in which to overexpress Msx2, which would have a different genetic background to the breast cell lines used in this study and may therefore have altered downstream responses to Msx2 overexpression. This variation in the cellular response to Msx2 indicates that the context of Msx2 overexpression, as well as its subcellular localisation and intensity, is an important factor to consider when examining the role of Msx2 in breast tumours. In addition, the intracellular milieu of transcriptional regulators and cofactors may play an important role in regulating the downstream effects of Msx2 overexpression.

The varying effect of Msx2 expression in different tumour types also suggests a cell type-specific effect. Indeed, Msx2 has been associated with high tumour grade in pancreatic cancer [17]. Studies in breast cancer have suggested a link to EMT through induction of Cripto-1 and the c-Src pathway [16], although this is contradicted by studies showing that Msx2 downregulates the prometastatic factor BSP [18]. The reasons for the context-specific effects of Msx2 overexpression are yet to be explained and may be a result of both changes in cell lineage and protein localisation. However, the results from our clinical study make it clear that Msx2 is a marker of good prognosis in human breast cancer, illustrating the principle that data obtained from *in vitro *laboratory work must be supplemented by clinical investigation to determine the real prognostic value of a biomarker.

## Conclusions

These findings indicate that increased expression of both Msx2 mRNA and protein are associated with improved patient outcome in breast cancer. Furthermore, we have shown that ectopic expression of Msx2 in breast cell lines leads to the induction of apoptosis and radically decreased cell viability. This study suggests that increased Msx2 results in improved outcome for breast cancer patients, possibly by increasing the likelihood of tumour cell death by apoptosis.

## Abbreviations

BCSS: breast cancer-specific survival; BMP: bone morphogenetic protein; BSP: bone sialoprotein; CPA: cell pellet array; DLX5: distal-less homeobox 5; EMT: epithelial to mesenchymal transition; ER: estrogen receptor; ERK: extracellular signal-regulated kinase; EV: empty vector; FFPE: formalin-fixed paraffin-embedded; Her2: human epidermal growth factor 2; HR: hazard ratio; IHC: immunohistochemistry; Ki67: proliferation-related Ki-67 antigen; Msx2: muscle segment homeobox 2; MTT: 3-(4,5-Dimethylthiazol-2-yl)-2,5-diphenyltetrazolium bromide; OS: overall survival; PBS: phosphate-buffered saline; PI: propidium iodide; PR: progesterone receptor; RBM15: RNA-binding motif protein 15; RFS: recurrence-free survival; Shh: sonic hedgehog-1; SPEN: a derivation of 'split ends' (*Drosophila*); SPOC: SPEN paralog and ortholog C-terminal; TMA: tissue microarray; VEGF: vascular endothelial growth factor.

## Competing interests

The authors declare that they have no competing interests.

## Authors' contributions

FL participated in the design of the study, carried out the transcriptomic dataset mining, antibody validation, TMA staining, lentiviral overexpression, and functional analysis; performed the statistical analysis, and drafted the manuscript. GG participated in the design of the lentiviral study, and designed and validated the lentiviral construct used in this study. RH and FL scored the immunohistochemical staining on the TMA. DB, FM and WG participated in the design of the study and edited the manuscript. KJ constructed the TMAs and collated the clinical information. All authors read and approved the final manuscript.

## Supplementary Material

Additional file 1 **Supplementary data**. Analysis of the available and missing cohorts for the Msx2 study from the original cohort of 512 patients on the TMA. Click here for file

Additional file 2 **Figure S1: Automated image analysis of Msx2 protein expression**. Kaplan-Meier estimates of Overall Survival stratified according to (a) Msx2 cytoplasmic and (b) Msx2 nuclear expression, based on automated image analysis data. Click here for file
