# Supplementary material for: Homeobox transcription factor muscle segment homeobox 2 (Msx2) correlates with good prognosis in breast cancer patients and induces apoptosis in vitro
Source: Breast Cancer Res. 2010 Aug 3;12(4):R59. doi: 10.1186/bcr2621 (PMC2949651; doi:10.1186/bcr2621)
Supplement: Additional file 1 — Supplementary data. Analysis of the available and missing cohorts for the Msx2 study from the original cohort of 512 patients on the TMA. [file bcr2621-S1.DOC]

**Additional data file 1:**

**Analysis of the available and missing cohorts for the Msx2**

**study from the original cohort of 512 patients on the TMA**

| **Variable** | **Cytoplasmic Msx2** | | |
| --- | --- | --- | --- |
|  | Msx2 available  (n=281) | Msx2 missing  (n=231) | p-value |
| Age  ≤ 50  >50 | 37 (13.2)  244 (86.8) | 43 (18.6)  188 (81.4) | 0.091 |
| Tumor size  ≤ 2cm  > 2cm | 180 (64.1)  101 (35.9) | 141 (61)  90 (39) | 0.482 |
| Histological type  Ductal  Lobular  Tubular  Medullary  Mucinous  Unknown | 190 (73.1)  41 (15.8)  17 (6.5)  5 (1.9)  7 (2.7)  21 | 149 (68.3)  32 (14.7)  17 (7.8)  10 (4.6)  10 (4.6)  13 | 0.336* |
| Nodal status  Negative  Positive  Unknown | 158 (63.2)  92 (36.8)  31 | 133 (64.3)  74 (35.7)  24 | 0.816 |
| Tumor grade  I  II  III  Unknown | 69 (24.6)  117 (41.8)  94 (33.6)  1 | 58 (25.2)  96 (41.7)  76 (33)  1 | 0.986 |
| ER status  Negative  Positive  Unknown | 39 (14.3)  233 (85.7)  9 | 33 (15.9)  174 (84.1)  24 | 0.627 |
| PR status  Negative  Positive  Unknown | 85 (38.3)  137 (61.7)  59 | 56 (37.8)  92 (62.2)  83 | 0.930 |
| Ki 67 (%)  < 10%  >10%  Unknown | 108 (39.9)  163 (60.1)  10 | 74 (36.6)  128 (63.4)  29 | 0.477 |
| VEGF (%)  Low (0-2+)  High (3)  Unknown | 169 (81.6)  38 (18.4)  74 | 104 (75.4)  34 (24.6)  93 | 0.160 |
| Her2 (%)  Low (0-2)  High (3)  Unknown | 236 (90.4)  25 (9.6)  20 | 188 (90.4)  20 (9.6)  23 | 0.989 |
| Cyclin D1 (%)  Low (0-1%)  Med (2-25%)  High (>25%)  Unknown | 37 (13.8)  58 (21.6)  173 (64.6)  13 | 32 (16.7)  42 (21.9)  118 (61.5)  39 | 0.676 |

*Fisher’s exact test;, otherwise, χ2 test
