# Supplementary figures and images for: Homeobox transcription factor muscle segment homeobox 2 (Msx2) correlates with good prognosis in breast cancer patients and induces apoptosis in vitro
Source: Breast Cancer Res. 2010 Aug 3;12(4):R59. doi: 10.1186/bcr2621 (PMC2949651; doi:10.1186/bcr2621)

**(a) CYTOPLASMIC**

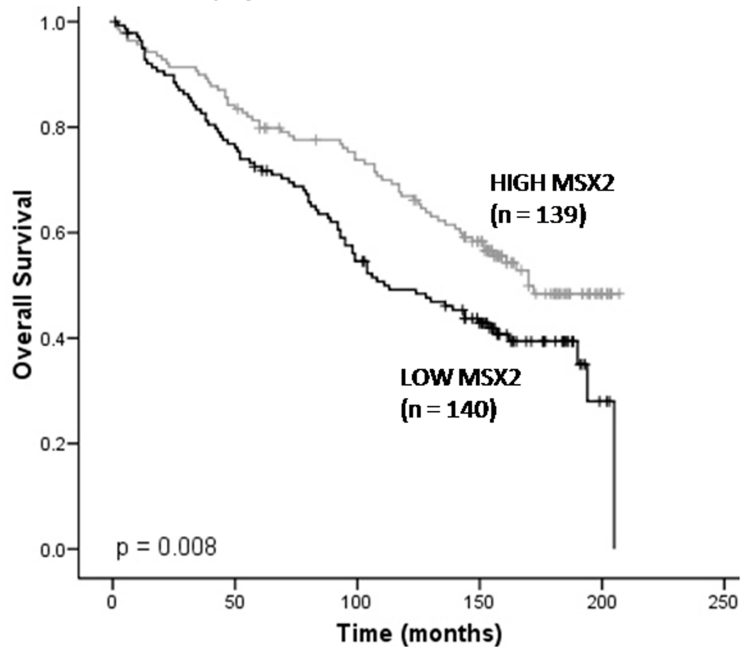

**(b) NUCLEAR**

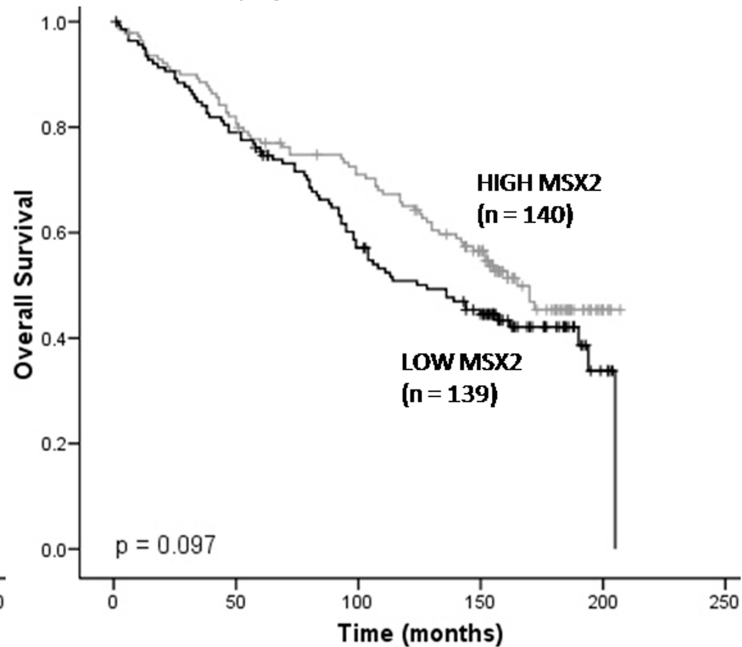

Supplement: Additional file 2 — Figure S1: Automated image analysis of Msx2 protein expression. Kaplan-Meier estimates of Overall Survival stratified according to (a) Msx2 cytoplasmic and (b) Msx2 nuclear expression, based on automated image analysis data. [file bcr2621-S2.PDF]
